# Supplementary material for: Risk assessment of 2024 cattle H5N1 using age-stratified serosurveillance data
Source: Emerg Microbes Infect. 2025 Apr 22;14(1):2497304. doi: 10.1080/22221751.2025.2497304 (PMC12064101; doi:10.1080/22221751.2025.2497304)
Supplement: 4_Supple_Figures.docx [file TEMI_A_2497304_SM7664.docx]

**Supplementary Materials**


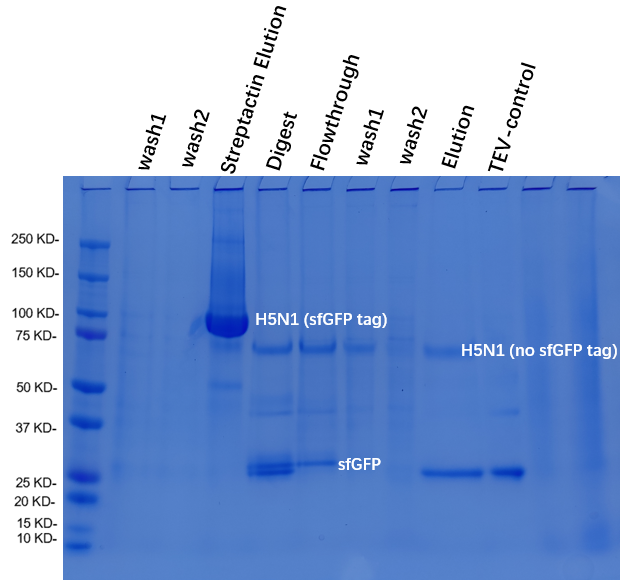


**Supplementary Figure S1.** The purified H5N1 protein obtained from the affinity column was analyzed using non-reducing (-DTT) SDS-PAGE. The streptactin was able to isolate H5N1 with sfGFP tag. After TEV digestion, H5N1 without the sfGFP tag was obtained using HisSep Ni-NTA Agarose Resin. The band corresponding to H5N1 without the sfGFP tag is significantly lower than that of H5N1 with the sfGFP tag.


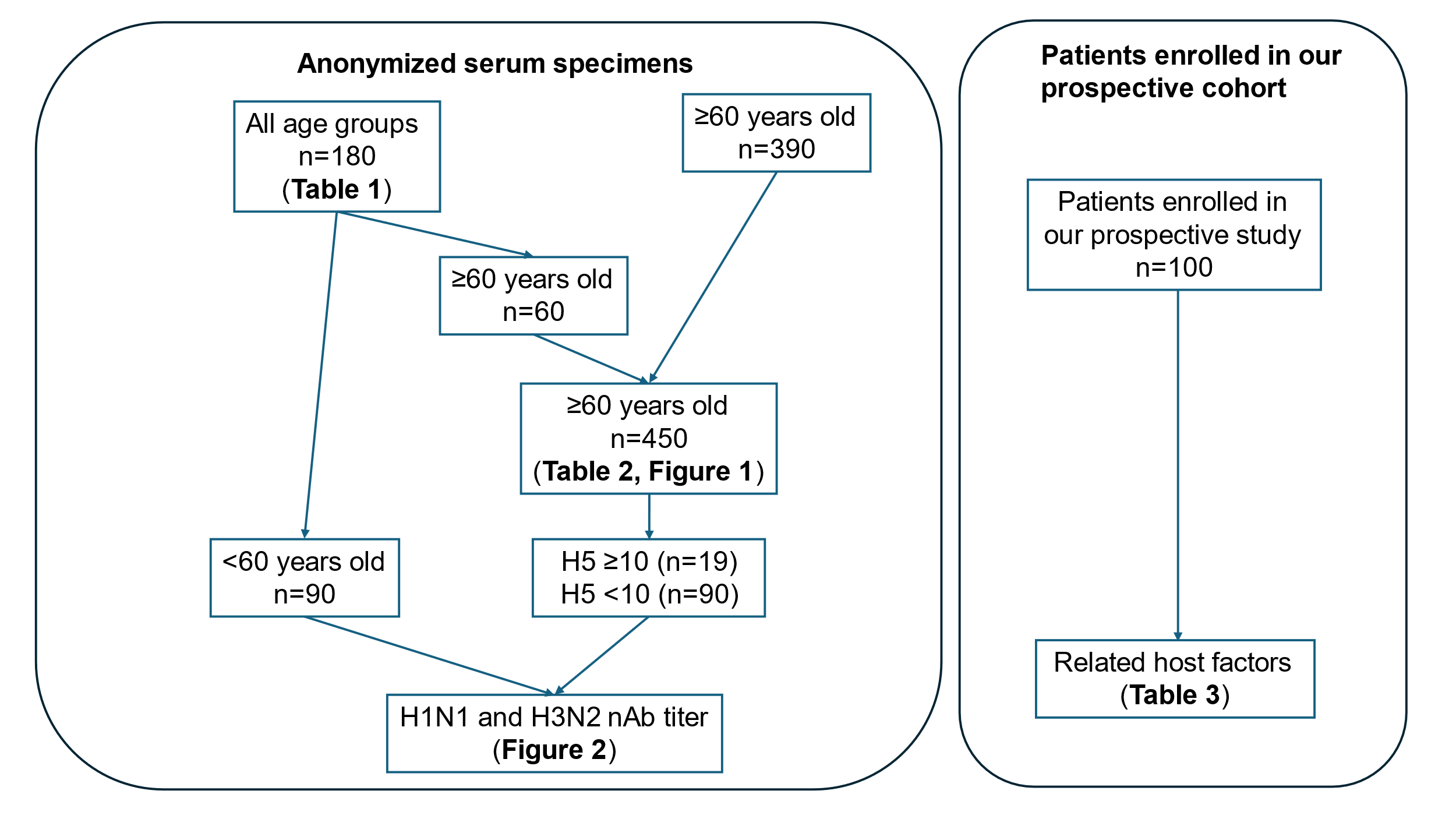


**Supplementary Figure S2.** Schematic diagram showing the number of specimens for all tables and figures.
